# Cellular and Molecular Defects Underlying Invasive Fungal Infections—Revelations from Endemic Mycoses

**DOI:** 10.3389/fimmu.2017.00735

**Published:** 2017-06-28

**Authors:** Pamela P. Lee, Yu-Lung Lau

**Affiliations:** ^1^LKS Faculty of Medicine, Department of Paediatrics and Adolescent Medicine, Queen Mary Hospital, The University of Hong Kong, Hong Kong, China; ^2^Shenzhen Primary Immunodeficiencies Diagnostic and Therapeutic Laboratory, The University of Hong Kong-Shenzhen Hospital (HKU-SZH), Shenzhen, China

**Keywords:** endemic mycoses, primary immunodeficiencies, human immunodeficiency virus, histoplasmosis, coccidioidomycosis, paracoccidioidomycosis, blastomycosis, *Taloromyces marneffei*

## Abstract

The global burden of fungal diseases has been increasing, as a result of the expanding number of susceptible individuals including people living with human immunodeficiency virus (HIV), hematopoietic stem cell or organ transplant recipients, patients with malignancies or immunological conditions receiving immunosuppressive treatment, premature neonates, and the elderly. Opportunistic fungal pathogens such as *Aspergillus, Candida, Cryptococcus, Rhizopus*, and *Pneumocystis jiroveci* are distributed worldwide and constitute the majority of invasive fungal infections (IFIs). Dimorphic fungi such as *Histoplasma capsulatum, Coccidioides* spp., *Paracoccidioides* spp., *Blastomyces dermatiditis, Sporothrix schenckii, Talaromyces (Penicillium) marneffei, and Emmonsia* spp. are geographically restricted to their respective habitats and cause endemic mycoses. Disseminated histoplasmosis, coccidioidomycosis, and *T. marneffei* infection are recognized as acquired immunodeficiency syndrome (AIDS)-defining conditions, while the rest also cause high rate of morbidities and mortalities in patients with HIV infection and other immunocompromised conditions. In the past decade, a growing number of monogenic immunodeficiency disorders causing increased susceptibility to fungal infections have been discovered. In particular, defects of the IL-12/IFN-γ pathway and T-helper 17-mediated response are associated with increased susceptibility to endemic mycoses. In this review, we put together the various forms of endemic mycoses on the map and take a journey around the world to examine how cellular and molecular defects of the immune system predispose to invasive endemic fungal infections, including primary immunodeficiencies, individuals with autoantibodies against interferon-γ, and those receiving biologic response modifiers. Though rare, these conditions provide importance insights to host defense mechanisms against endemic fungi, which can only be appreciated in unique climatic and geographical regions.

## Introduction

Endemic mycoses are infections caused by a diverse group of fungi that occupy specific ecologic niche in the environment (1). The major pathogenic fungi in this group, including *Blastomyces dermatiditis, Coccidioides immitis* and *C. posadasii, Paracoccidioides brasiliensis* and *P. lutzii, Histoplasma capsulatum, Sporothrix schenckii, Talaromyces marneffei* (formerly known as *Penicillium marneffei*) and *Emmonsia* spp., belong to the phylum *Ascomycota* and are evolutionary related (2) (Table 1). They share the common characteristic of thermal dimorphism—they grow as saprophytic molds in the environment at temperatures ranging from 25 to 30°C, and undergo morphological switch to the yeast form, or spherules in *Coccidioides*, at body temperatures of mammalian hosts. The yeast form serves to accommodate intracellular growth within host phagocytes (3). Majority of these organisms are primary pathogens that are able to cause disease in healthy human individuals. However, they may cause severe, disseminated infections in immunocompromised hosts, such as patients with human immunodeficiency virus (HIV) infection, organ transplant, or hematopoietic stem cell transplant (HSCT) recipients, and those with autoimmune disorders receiving immunosuppressants (4–7). In particular, *T. marneffei* and *Emmonsia* spp. more typically cause disease in HIV-infected individuals (5–8). The HIV pandemic and the increasing use of immunosuppressive medications, such as calcineurin and tumor necrosis factor (TNF) inhibitors, have resulted in a rising trend of histoplasmosis and coccidioidomycosis in endemic regions (9, 10). Exposure to the specific environmental niche, either residential, occupational, or travel precedes the development of disease.

**Table 1:** Endemic regions, natural habitats and risk factors of exposure to endemic mycoses.

	Phylum	Order	Endemic regions	Animal hosts	Disease in human
*Coccidioides immitis, C. posadasii*	Ascomycota	Onygenales	Southwestern USA, northern Mexico, Central and South America	Non-human primates, domesticated or wide mammals, dogs, cats, horses, llamas, snakes	Coccidioidomycosis
*Paracoccidioides brasiliensis, P. lutzii*	Ascomycota	Onygenales	South America	Domesticated and wild animals (monkeys and armadillos), dogs	Paracoccidioidomycosis
*Histoplasma capsulatum*	Ascomycota	Onygenales	Worldwide; hyperendemic in Mississippi and Ohio river valleys in USA	Cattle, sheep, horses	Histoplasmosis
*Blastomyces dermatitidis*	Ascomycota	Onygenales	Worldwide (endemic in North America, autochthonous in Africa, South America, and Asia)	Dogs, cats, horses, marine mammals	Blastomycosis
*Emmonsia* spp.	Ascomycota	Onygenales	South Africa	Wild rodents	Emmonsiosis
*Sporothrix schenckii, S. brasiliensis*	Ascomycota	Ophiostomales	Worldwide	Cats, occasionally dogs, horses, cows, goats, mules, pigs, rats, armadillos, camels, dolphins, birds	Sporotrichosis
*Talaromyces (Penicillium) marneffei*	Ascomycota	Eurotiales	Southwest and southern China; Southeast Asia	Bamboo rats, domestic animals such as dogs and cats	Penicilliosis

## Geographical Distribution of Endemic Mycoses

**Figure 1:**
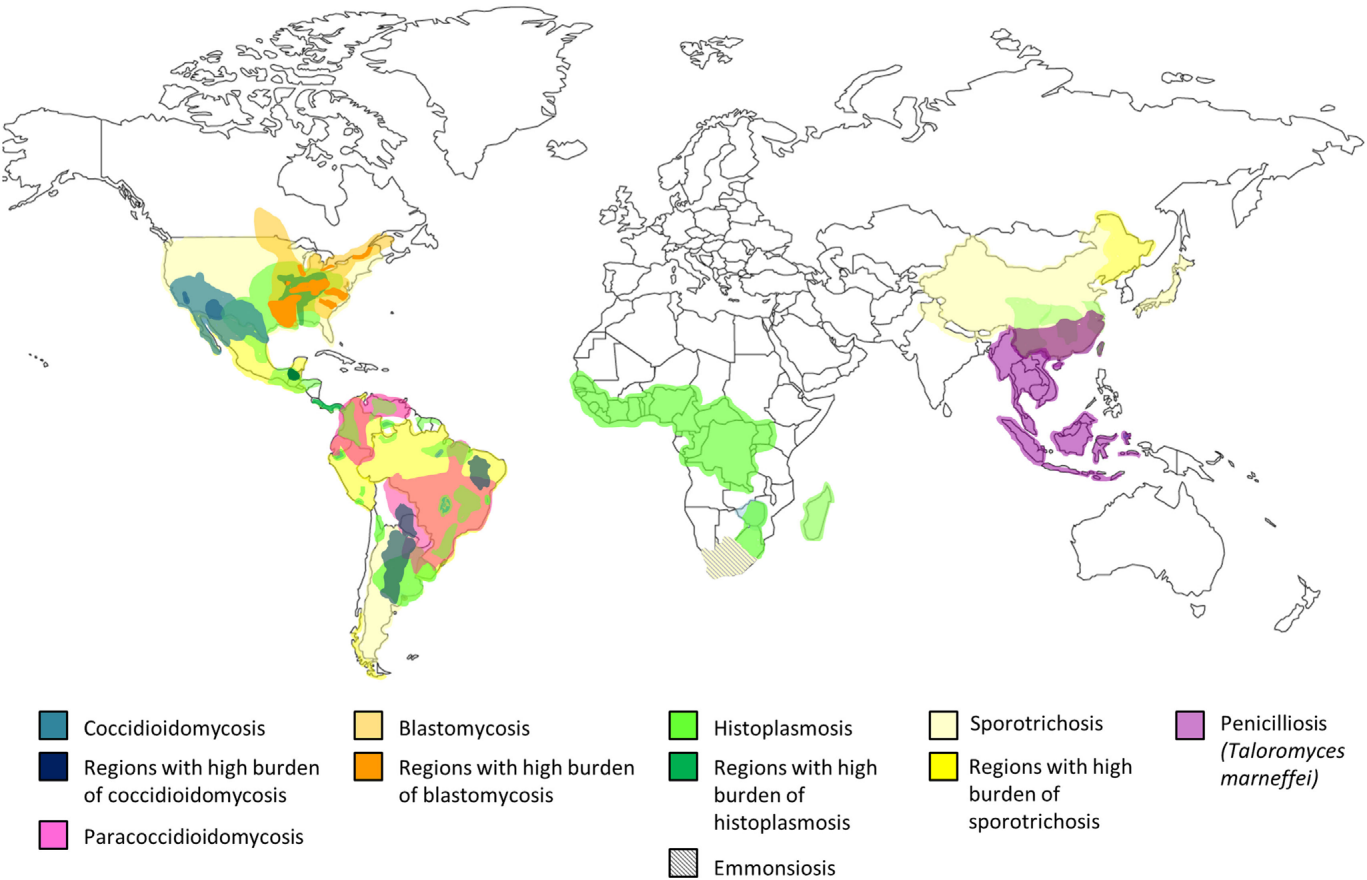
Global distribution of endemic mycosis (1, 13, 14, 18–20, 27, 28, 32–39).

Endemic mycoses occur predominantly in specific climate zones. Coccidioidomycosis is present in semidesert areas, histoplasmosis and paracoccidioidomycosis (PCM) are prevalent in tropical regions while *T. marneffei* is endemic in subtropical regions, and blastomycosis belongs to temperate climates (6–8). Coccidioioidomycosis (11–13) and histoplasmosis (14–18) are widely distributed in the American continent and some tropical regions, while PCM is limited to Central and South America (19–21). *T. marneffei* is unique to Southeast Asia (22–24), and blastomycosis is found in North America, and Central and East Africa (25–27). *S. schenckii* is distributed around the world, mainly reported in those tropical and temperate zones with high humidity and mild temperatures (22–27°C) (28). Recently, an emerging thermally dimorphic fungus within the genus *Emmonsia* that is most closely related to *E. pasteuriana* has been recognized to be uniquely associated with in HIV infection in South Africa (29–31). The geographical distribution of endemic mycoses is shown in Figure 1 (1, 12–14, 18–20, 27, 28, 32–39).

The biological niche is specific for each endemic fungus and knowledge of their natural habitat provides understanding about the risk factors for exposure to these pathogens (Table 2). *C. immitis* and *C. posadasii* are saprophytic fungi, which exist in their mycelial form in dry, alkaline soil in deserts with very low precipitation and extreme temperature variations (11–13). Coccidioidomycosis is most prevalent in Arizona and California in the United States (US) (40, 41). In contrast, *H. capsulatum* thrive in the tropical zones with high relative humidity, and its growth is favored by soil contaminated by bird and chicken excreta or bat guano, which creates an environment with high nitrogen content (14, 15). *P. brasiliensis* is also found in the tropical and very humid regions, especially in acidic soil where coffee and sugar canes are cultivated (20). PCM is prevalent in South America (Brazil, Columbia, Venezuela, Paraguay) and some regions of Central America and Mexico (19–21). *B. dermatitidis* exists in wet soils, and the most significant endemic epicenter is in Eastern US between the Ohio and Mississippi River valleys (25–27). *T. marneffei* is highly endemic in Thailand, Vietnam, Southern China, and other subtropical areas in Southeast Asia (22–24). Bamboo rats (*Rhizomys* spp. and *Cannomys* spp.) and soil from their burrows are important enzootic and environmental reservoirs of *T. marneffei*, respectively (39). *S. schenckii* is found in the soil containing decaying vegetation such as dead wood, mosses, hay, and cornstalks. Sporotrichosis is also widely prevalent in warm-blooded animals including cats, dogs, armadillos, birds, and parrots, which constitute a source of zoonotic transmission (28). To date, disseminated emmonsiosis associated with HIV infection caused by the new *Emmonsia* spp. has only been described in South Africa (29–31).

**Table 2:** Endemic regions, natural habitats, and risk factors of exposure to endemic mycoses.

	Main endemic regions	Other areas	Natural habitat	Human activities/conditions associated with increased risk of exposure	Occupations associated with increased risk of exposure
Coccidioidomycosis	Arizona and California in the US	Other parts of Southwestern US: New Mexico, Nevada, Utah and Texas Central America: Mexico, Guatemala, HondurasSouth America: Venezuela, Brazil, Argentina, Paraguay	Alkaline soils in dry desert climates	Soil excavations Dust storms, earthquakes	Construction site workers, farmers, military personnel, excavators, archeologists, inmates, and officers in correctional facilities
Histoplasmosis	*Histoplasma capsulatum var. capsulatum*: Ohio and Mississippi River Valleys in the Upper Midwest and Southeastern US *H. capsulatum var. duboisii* (African histoplasmosis): between 20° North and 20° South of the equator, and Madagascar	Southern Mexico Central and South America, e.g., Brazil, Uruguay, Paraguay, Argentina, Venezuela Mainland China: provinces along the Yangtze River (Yunnan, Sichuan, Hubei, Hunan, Jiangsu, Zhejiang) Southeast Asia, e.g., Thailand India, especially West Bengal and Uttar Pradesh along the Gangetic plains Europe: Italy (Po River Valley), Spain, Germany	Soil contaminated by bird and chicken excreta, or bat guano; bat caves	Walking on contaminated grounds, setting up tents Excavation, clearing foliage in a bird-roosting site	Miners, cave explorers, guano workers, farmers, beekeepers, archeologists
Paracoccidioidomycosis	*Paracoccidioides brasiliensis*: Brazil, Columbia, Venezuela, Paraguay *P. lutzii*: Center-West of Brazil	Central America and Mexico	Acid soils in area of coffee and sugar cane plantations	Soil exposure	Farmers, outdoor workers Women are less likely to develop clinical disease as estrogens inhibit conidial transformation to yeast cells
Blastomycosis	US: Mississippi and Ohio River valleys, Midwestern states Canada: provinces that border the Great Lakes and the Saint Lawrence Riverway, including Manitoba and northwestern Ontario	Middle and East Africa India	Warm, moist soil with high organic content, e.g., animal droppings	Occupational, residential, or recreational exposures to wildlife, soil, or bodies of freshwater	Occupational, residential, or recreational exposures that occur in close proximity to bodies of freshwater
*Talaromyces marneffei* infection	Thailand, Vietnam, Southern China	Laos, Malaysia, Myanmar, Cambodia, Hong Kong, Taiwan, Northeastern India	Soil, particularly burrows of bamboo rats	Soil exposure during rainy season	Agricultural workers
Sporotrichosis	Peru, Brazil, Mexico (Jalisco and Puebla mountain ranges)	Worldwide distribution in temperate and tropical regions—US, Asia (China, India, Japan), Australia	Soil and decaying vegetation, e.g., dead wood, sphagnum moss, cornstalks, hay	Cutaneous trauma with wound contamination by plants or soil; contact with reeds after flooding, bites from mice, armadillos, squirrels, cats, and dogs	Farming, gardening, flower vending, handling hay, animal husbandry, armadillo hunting (in Uruguay), mining

## Pathogenesis and Clinical Manifestations

The acquisition of endemic fungi relates to human activities and climatic conditions that increase the risk of exposure to these organisms in susceptible individuals. Coccidioidomycosis, PCM, histoplasmosis, blastomycosis, and *T. marneffei* infection are acquired by the respiratory route. Occupational or recreational activities causing disturbance of the soil environment lead to aerosolization of the conidia, which could then be inhaled by exposed individuals to cause infection (1, 6–8, 14, 20, 22, 23, 27). In contrast, inhalation is not the major route by which *S. schenkii* is acquired. Instead, it typically occurs after traumatic inoculation or through microscopic breaks in the skin caused by pricks with plants, although the mode of transmission was not obvious in 60% of patients with sporotrichosis. Infection of the skin and subcutaneous tissues develops at the site of penetrating trauma and may spread to the muscles, fascia, cartilage, and bones (42, 43). The clinical features of these endemic mycoses are summarized in Table 3.

**Table 3:** Clinical manifestations of endemic mycoses and risk factors for disseminated disease.

	Asymptomatic infections	Sites of initial infection	Distant spread/disseminated disease	Conditions predisposing to disseminated disease
Coccidioidomycosis	Asymptomatic infections in majority of immunocompetent individuals	Pneumonia, often as mild respiratory illness Rarely primary cutaneous lesions at the site of inoculation due to injury	Fungemia, lymphadenopathy, skin lesions (in the vicinity of infected lymph nodes manifesting as abscesses, ulcers, gummata, retracting scars), osteoarticular involvement, meningitis	HIV infection PID Patients on anti-TNF-alpha monoclonal antibodies Chemotherapy, organ transplant and HSCT, immunosuppressants Diabetes mellitus, cardiopulmonary disease, pregnancy Higher risk of dissemination in African-American and Filipino
Histoplasmosis	Mostly acquired during childhood as asymptomatic infection	Most are self-limiting Acute pulmonary histoplasmosis: fever, cough, dyspnea, enlarged mediastinal, or hilar lymph nodes Chronic pulmonary histoplasmosis: cavitating lung lesions Rarely primary cutaneous lesions by injury—chancre, lymphangitis, nodular gummata	Fungemia, hepatomegaly, splenomegaly, bone marrow involvement, pancytopenia, reactive hemophagocytosis, oropharyngeal ulcers, gastrointestinal bleeding, endocarditis, skin lesions (molluscum-like papules, nodular/gummatous lesions), meningeal involvement, adrenal (Addison’s disease)	HIV infection PID Patients on anti-TNF-alpha monoclonal antibodies Chemotherapy, organ transplant, and HSCT, immunosuppressants
Paracoccidioidomycosis	Asymptomatic infections in majority of immunocompetent individuals	Juvenile form: generalized lymphadenopathy, hepatosplenomegaly, lesions in the skin, oral and intestinal mucosa, bone involvement Chronic (“adult”) form: pneumonia, mucosal lesions in the oropharyngeal or nasal region, palatal ulceration extending to the gums and tongue	Involvement of the digestive tract, pancreas and adrenal glands; hepatomegaly, splenomegaly	HIV infection PID
Blastomycosis	Asymptomatic infections in majority of immunocompetent individuals	Pneumonia Rarely primary skin involvement at the site of inoculation due to injury, manifesting as lymphangitis, ulcers, nodules, verruca	Skin involvement (nodules, gummata, abscesses, ulcers)	Uncommon association with acquired immunodeficiencies; no case of PID identified in individuals with blastomycosis
*Talaromyces marneffei* infection	Asymptomatic infections in majority of immunocompetent individuals	Localized skin disease due to direct inoculation Lymphadenitis Pneumonia	Fungemia, pneumonia, hepatomegaly, splenomegaly, lymphadenopathy, bone marrow involvement, osteoarticular involvement, cutaneous lesions, neurological manifestations	HIV infection PID Individuals with autoantibodies against IFN-gamma Splenectomy, diabetes mellitus, autoimmune disease Chemotherapy, organ transplant and HSCT, immunosuppressants Novel anti-cancer target therapies, e.g., anti-CD20 monoclonal antibodies, kinase inhibitors
Sporotrichosis	Most cases are acquired through traumatic implantations, often with spontaneous resolution	Skin infections may progress into chronic cutaneous, subcutaneous, or deeper infections involving the lymphatics, fascia, muscles, cartilage and bones	Occasional cases of pulmonary or disseminated disease: multiple skin lesions at non-contiguous sites, mucosal (nasal, oral, conjunctival), osteoarticular, pulmonary and meningeal involvement	HIV infection PID Chemotherapy, organ transplant and HSCT, immunosuppressants Diabetes mellitus, alcoholism, cirrhosis, malnutrition

*HIV, human immunodeficiency virus; HSCT, hematopoietic stem cell transplant; IFN, interferon; TNF, tumor necrosis factor; PID, primary immunodeficiencies*.

After entry to the body, inhaled conidia converts to the yeast form, which are taken up by tissue-resident macrophages. In most individuals, the pathogenic yeasts can be eliminated by the macrophages and the infection is usually asymptomatic or mild, and self-limiting in most cases. In patients whose immunity is compromised, the yeasts continue to proliferate in the macrophages, which if uncontained, systemic dissemination may occur *via* the reticuloendothelial system (12, 23, 44–46). HIV infection is the most important risk factor for disseminated endemic mycoses (5). Other risk factors include malignancy and immunosuppression, as listed in Table 3 (4–7, 9, 10).

## Endemic Mycoses: Insights from the HIV Epidemic

Human immunodeficiency virus infection is the most common cause for disseminated or extrapulmonary forms of histoplasmosis, coccidioidomycosis, and *T. marneffei* infection, particularly in patients with profound T-cell lymphopenia (CD4+ lymphocytes <200/μL) (12, 47–50). They are considered as acquired immunodeficiency syndrome (AIDS)-defining conditions in the World Health Organization (WHO) clinical staging of HIV/AIDS for adults and adolescents (51). The association between PCM and AIDS is relatively rare in contrast to the higher incidence of other systemic mycosis. HIV coinfection has been detected in about 5% of patients with PCM (52, 53). Although PCM and sporotrichosis are not included as AIDS-defining conditions, they are increasingly recognized as an emerging neglected opportunistic infections in HIV patients in Latin America (7, 53, 54). Systemic sporotrichosis with organ involvement or widespread cutaneous lesions may occur in these patients, causing a mortality rate of up to 30% (54, 55). Blastomycosis infrequently develops in HIV patients, but disease tends to be more severe with increased risk of central nervous system (CNS) involvement with high mortality (46, 56). The new species of *Emmonsia* spp. discovered in South Africa was found to cause disseminated infection almost exclusively in patients with AIDS (29–31).

The first case series of histoplasmosis in HIV-infected patients was described in 1982 in the US (57). Extrapulmonary or disseminated histoplasmosis became an AIDS-defining disease in 1987 (58). The increase in morbidity and mortality from histoplasmosis has been largely contributed by the HIV pandemic. In the US, the availability of highly active anti-retroviral therapy (HAART) and lipid formulations of amphotericin B, the increased awareness of the disease, and the development of rapid, non-invasive diagnostic methods led to decrease in the incidence and mortality associated with histoplasmosis in patients with AIDS (16). On the other hand, extrapulmonary or disseminated histoplasmosis is becoming an important health issue in the increasing number of patients receiving chemotherapy, solid organ or HSCT, immunosuppressive treatment especially TNF-α blockade (59), as well as a rare group of patients with primary immunodeficiencies (PIDs). In contrast, in endemic areas of Latin America histoplasmosis occurs in up to 25% of HIV-infected patients and represents the first manifestation of AIDS in up to 50–75% of patients (60, 61). Due to the lack of diagnostic facilities and algorithm, histoplasmosis is undiagnosed in many HIV-infected patients and is considered as an “invisible burden” of AIDS in less resourced countries (62, 63). These phenomena illustrate how demographics of histoplasmosis could be shaped by HIV burden and socioeconomic forces in different endemic regions.

Shortly after the description of increased susceptibility to histoplasmosis in AIDS, *Coccidioides* infection emerged as an important form of mycosis in patients with HIV infection (64–66). A prospective study at an Arizona HIV clinic in 1988 showed a cumulative incidence of active coccidioidomycosis of 25% during 41 months of follow-up, corresponding to an average annual incidence of 7.3% (67). In contrast, in a retrospective review at the same clinic during 2003–2008, only 11.3% of HIV-infected patients (*n* = 257) had coccidioidomycosis, and the annual incidence was only 0.9% when compared to the previous study (47). Both studies showed that CD4 count was the only predictor for developing active coccidioidomycosis; factors such as a history of coccidioidomycosis and duration of residence in an endemic area or age, sex, race, ethnicity, plasma HIV RNA level, or receipt of HAART were not associated with increased risk for coccidioidomycosis (11, 47, 67).

While histoplasmosis and coccidioidomycosis are well recognized as human pathogens long before the HIV epidemic, the importance of *T. marneffei* as a human disease was not recognized until the outbreak of HIV in Asia (22, 68, 69). In 1973, the first naturally occurring human case of *T. marneffei* infection was reported in an American minister with Hodgkin’s disease who had been residing in Southeast Asia (70). No more than 50 cases were reported in the literature during the early 1980s (68–76). From 1988, *T. marneffei* infection was increasingly observed in patients with advanced HIV infection, initially in foreign visitors who have been to endemic regions, and later in local residents who were native to endemic parts of Thailand and Vietnam (22, 23, 77–79). In Northern Thailand, *T. marneffei* infection is the third most common opportunistic infection, accounting for 15–20% of all AIDS-related illness, after tuberculosis (TB) and cryptococcosis. *T. marneffei* infection is estimated to occur in 2.3% of new AIDS cases, compared with 0.39% for histoplasmosis (80). The trend of *T. marneffei* infection closely paralleled that of HIV, and in areas where reduction of HIV transmission and availability of HAART have improved, a decrease in the prevalence of *T. marneffei* infection has been observed (23, 81).

Together with *Emmonsia* spp., which causes disseminated infection almost exclusively in advanced HIV infection in South Africa (29–31), it is apparent that the epidemiology of coccidioidomycosis, histoplasmosis, and *P. marneffei* evolves with HIV epidemic. The close relationship between disease manifestation and severity with CD4+ cell count confirms the central importance of cell-mediated immunity against endemic fungi.

## PIDs in Endemic Mycoses: Needles in a Haystack?

Primary immunodeficiencies are rare inborn errors of immunity. Defects of T-cell development and differentiation, phagocytic functions, and pathways involved in the innate recognition of pathogens and downstream signaling are associated with increased risk of fungal infections, the most common being candidiasis and aspergillosis. Endemic mycoses are rarely described in patients with PID, and little is known about the spectrum of PID associated with increased susceptibility to endemic fungi. On the other hand, as individuals who are apparently healthy can also develop disease caused by endemic fungi, recognition of those who may have an underlying PID could be a challenge.

*Talaromyces marneffei* infection is mostly seen in advanced HIV infection with CD4+ cell count <100/μL, and in fact, up to 80% or more of the cases have CD4+ count <50/μL (22, 81, 82). Only small proportion of disseminated *T. marneffei* infection occurs in patients with secondary immunodeficiencies (22, 23, 83, 84). It is otherwise rare in healthy persons, especially in children. The close epidemiological relationship between HIV and *T. marneffei*, and the fact that *T. marneffei* is an AIDS-defining illness (51) suggests that individuals who are HIV negative and without secondary immunodeficiencies may have underlying immune defects that are unrecognized. A systematic review by Lee et al (85) on more than 500 articles published in English and Chinese from 1970 to 2011 on penicilliosis revealed 32 patients aged 3 months to 16 years with *T. marneffei* infection but without known HIV infection. Twenty-four patients (75%) had disseminated disease, and 55% died of *T. marneffei* infection. Eight patients had PID or blood disorders, while four others had abnormal immune functions. Immune evaluations of the remaining patients were unstated. This observation highlights the knowledge gap in the immunological susceptibility to *T. marneffei*.

Two systematic reviews on PID in histoplasmosis and coccidioidomycoses were recently published. Lovell et al. (86) summarized all published cases of histoplasmosis in patients with PID up to August 2015 and revealed 47 patients with underlying PID, defined either molecularly or clinically. Together with the four patients described in their report, more than 50 PID patients have been documented to have *Histoplasma* infection. Disseminated histoplasmosis occurred in 68% of cases, and two deaths occurred because of progressive disease. Another systematic literature search on disseminated coccidioidomycosis yielded 370 case reports, and 8 cases of PID were identified (87). The frequency of PID underlying endemic mycoses is unknown. Given the rarity of PID, the proportion of PID accounting for disseminated endemic mycoses is likely to be small. However, the cellular and molecular defects of these PID can provide important mechanistic insights into host defense mechanisms against endemic fungi. More importantly, disseminated or extrapulmonary forms of endemic mycoses can be utilized as unique indicators for PIDs, which is of particular relevance to clinicians working in respective endemic areas (88).

## PIDs Underlying Fungal Infections

**Figure 2:**
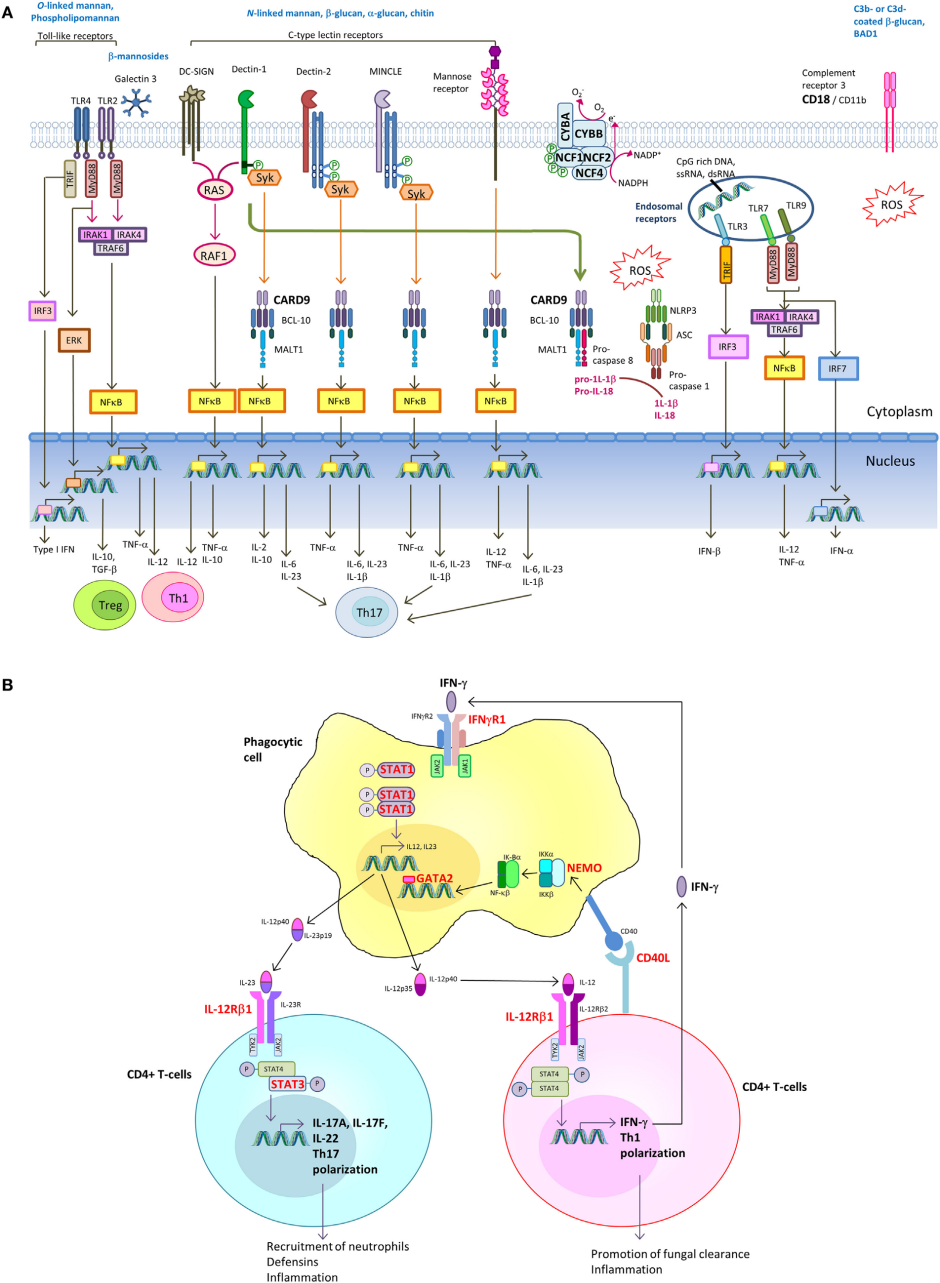
Signaling pathways in innate recognition of fungal pathogens and differentiation of CD4+ T helper cells. **(A)** Pathogen-associated molecular patterns (PAMPs) expressed by fungi are recognized by host pattern recognition receptors (PRRs), including toll-like receptors (TLRs), C-type lectin receptors (CLRs) [e.g., dendritic cell (DC)-specific ICAM3-grabbing non-integrin (DC-SIGN), Dectin-1, Dectin-2, MINCLE, and mannose receptor] and complement receptor 3 (CR3). TLRs and CLRs activate multiple intracellular signaling pathways upon binding to specific fungal PAMPs, including β-glucans, chitin, *O*-linked mannan and *N*-linked mannan, and nucleic acids. These signals activate canonical or non-canonical nuclear factor-κB and the NOD-, LRR- and pyrin domain-containing 3 (NLRP3) inflammasome. The integration of simultaneously activated PRRs occurs at the level of intracellular adaptors and transcription factors shared between overlapping pathways. The resulting cytokine responses shape the activation of adaptive immune response. Induction of IL-12 drives IFN-γ production by T-helper 1 (Th1) cells, which is crucial for phagocyte activation. Induction of IL-1β, IL-6, and IL-23 promotes Th17 differentiation. Regulatory T-cells (Treg) act as host-driven homeostatic response to keep inflammation under control. **(B)** Th1 and Th17 differentiation. Polarization of naive T cells into Th1 leads to IFNγ production, and its signaling is mediated through the Janus kinase (JAK)–signal transducer and activator of transcription 1 (STAT1) pathway, leading to transcription of IFNγ-inducible genes. IL-6 and IL-21 upregulate the expression of the retinoic acid-related orphan receptor RORγt and RORα, leading to expression of the inducible component of the IL-23 receptor (IL-23R) and further Th17 development. IL-17A and IL-17F produced by Th17 cells augments neutrophil production in the bone marrow and their recruitment to the site of infection. IL-17A, IL-17F, and IL-22 promote production of antimicrobial peptides in epithelial cells. Molecules in which genetic defects have been identified to be associated with increased susceptibility to endemic mycoses are marked in bold red.

Host immune response toward fungal pathogens is initiated by the recognition of invading fungi *via* pattern recognition receptors (PRRs) expressed on neutrophils, monocytes, macrophages, and dendritic cells (DCs). Receptor-mediated signaling induces downstream events such as cytokine and chemokine release, phagocytosis, and respiratory burst ultimately leading to fungal killing (89–93). In addition, the cytokine responses shape the induction of Th-1 and Th-17 adaptive immune response. IL-12 drives IFN-γ production by T-helper 1 (Th1) cells, which is crucial for phagocyte activation. On the other hand, IL-1β, IL-6, and IL-23 promotes Th17 differentiation (89–91). The various PRRs that recognize fungal pathogen-associated molecular patterns and the downstream signaling pathways leading to induction of Th1 and Th17 response are shown in Figure 2A.

Defects of the dectin-1/CARD9-MALT1-BCL10 signaling pathway are associated with chronic mucocutaneous candidiasis (CMC) (94). Patients with CARD9 deficiency have defects in Th17 differentiation and impaired neutrophil killing (95), and they are susceptible to CMC (96), deep dermatophytoses (97), and invasive fungal infection, particularly *Candida* meningitis. Other monogenic disorders causing CMC include autosomal recessive (AR) IL-17 receptor A (IL17RA), AR IL-17RC, AR ACT1, and autosomal-dominant (AD) IL-17F deficiencies (98). These patients display deficiency of IL-17F and IL-17A/F (*IL-17F* mutations) or dysfunctional responses to IL-17A, IL-17A/F, and IL-17F (*IL17RA, IL-17RC*, and *ACT1* mutations). In patients with autoimmune polyendocrinopathy-candidiasis-ectodermal dystrophy, high plasma titers of neutralizing autoantibodies against IL-17A, IL-17F, and IL-22 can be detected, as a result of the lack of AIRE expression in the thymus causing impaired T-cell tolerance (99). However, endemic mycoses have not been reported in patients with these genetic defects.

Opportunistic fungal infections are common in patients with severe combined immunodeficiencies (SCIDs) and phagocytic disorders. Infants with classical SCID often have recurrent or persistent mucosal and/or cutaneous candidiasis involving the orodigestive tract, genital area, nails, and skin. *Pneumocystis jiroveci* pneumonia (PCP) and invasive fungal infections (IFIs) including systemic candidiasis and aspergillosis are often life-threatening (100, 101). Interestingly, endemic mycoses have not been described in SCID babies, at least in the English literature. It may be due to the fact that it is uncommon for infants to be exposed to the natural habitats containing those endemic fungi, but further epidemiological data would be required to address this. Numerical and functional defects of phagocytes such as severe congenital neutropenia, chronic granulomatous disease (CGD), and leukocyte adhesion deficiency (LAD) are major groups of PIDs predisposing to systemic candidiasis and invasive aspergillosis (102–104). Filamentous fungi other than *Aspergillus* causing pulmonary infections in CGD include *Geosmithia argillacea* and *Trichosporon inkin*. Osteomyelitis can also be caused by rare non-*Aspergillus* filamentous fungi, including *Cladophialophora arxii, Inonotus tropicalis, Scedospoium apiospermum, Penicillium piceum*, and *P. variotii*. Cerebral abscesses caused by dematiaceous molds such as *Exophiala* spp., *Phaeoacremonium* spp., and *Alternaria* spp. have been reported (105–107). Interestingly, endemic mycoses have not been reported in CGD and LAD.

In the following sections, we highlight the spectrum of PIDs, which are associated with increased susceptibility to disseminated endemic mycoses. The genetic defects are summarized in Figure 2B.

### Combined Immunodeficiencies: CD40 Ligand, Nuclear Factor Kappa B (NF-κB) Essential Modulator (NEMO), and DOCK8 Deficiencies

Mucosal candidiasis is common in combined immunodeficiencies e.g. CD40 ligand (CD40L) deficiency, NEMO (IKBG) deficiency, IKBA gain-of-function (GOF) mutation and DOCK8 deficiency, in addition to a broad range of viral, bacterial, and IFI (102, 103). CD40L is expressed on activated T-cells and signals through NEMO/NF-κB to induce IL-12 production. CD40L deficiency, also known as hyper-IgM syndrome, is inherited in an X-linked recessive manner. Patients are susceptible to opportunistic infections including PCP, cryptosporidiosis, and mycobacterial infections due to impaired interaction between T-cells and antigen-presenting cells (APCs). In addition, the failure of B-cell immunoglobulin (Ig) isotype switching results in markedly low serum IgG and IgA, while IgM is elevated (108, 109). Disseminated and cutaneous forms of histoplasmosis have been reported in eight patients in patients with X-linked hyper-IgM disorder (Table 4). Five cases had disseminated histoplasmosis, while two had lymphadenitis and one had cutaneous involvement only (86, 110–115). All of them responded well to antifungal therapy, and only two patients had recurrent histoplasmosis (86, 111). One case of PCM was reported in Brazil (116). It was shown that mature DCs from patients with CD40L deficiency exhibited markedly reduced IL-12 and increased IL-10 production in response to *P. brasiliensis* and *C. albicans* compared with normal controls, and T cells had significantly reduced IFN-γ production when cocultured with their DCs, whereas IL-4 and IL-5 production was increased. In contrast, T-cell proliferation and generation of TGF-β and IL-17 were comparable with normal controls. These findings suggested that the absence of CD40L during monocyte/DC differentiation leads to functional DC abnormalities, which may contribute to the susceptibility to fungal infections in patients with CD40L deficiency (117). Four cases of *T. marneffei* infection were reported in CD40L deficiency (118–120). One patient who had disseminated *T. marneffei* infection had rapid deterioration due to late diagnosis and died, and CD40L deficiency was diagnosed after he passed away (120).

**Table 4:** Endemic mycoses in CD40 ligand deficiency.

	Genetic defect	Endemic fungal pathogen	Gender/age, residence	Clinical manifestations	Other infections and comorbidities	Treatment and outcome
Tu et al. (110)	Not stated	*Histoplasma* spp.	M/3 years, US	Disseminated histoplasmosis with esophageal ulcers and bone marrow involvement	Cyclical neutropenia and anemia	Not stated
Hostoffer et al. (111)	Not stated	*Histoplasma capsulatum*	M/19 years, US	Disseminated histoplasmosis with pulmonary infiltrates, pancytopenia and splenomegaly	Tongue and per-rectal ulcers	Treated with amphotericin B, recurrence due to poor compliance to itraconazole prophylaxis
Yilmaz et al. (112)	Not stated	*Histoplasma* spp.	M/5 years, Turkey	Facial lesions, cervical lymphadenopathy, bilateral pulmonary infiltration and bronchiectasis	Recurrent pulmonary infections	Treated with ketoconazole
Danielian et al. (113)	p.R11X	*H. capsulatum*	M/6 months, Argentina	Histoplasma lymphadenitis	PCP and parvovirus B19 infection, recurrent pneumonia, adenitis, anemia	Not stated
Dahl and Eggebeen (114)	Not stated	*Histoplasma* spp.	M/14 years, US	Disseminated histoplasmosis complicated by fungemia and macrophage activation syndrome	Recurrent sinopulmonary infections and neutropenia	Liposomal amphotericin B for 14 days followed by oral itraconazole; macrophage activation syndrome treated with steroid and anakinra with prompt improvement
Lovell et al. (86)	c.289-15T > A	*Histoplasma* spp.	M/6 years (patient 2)	Disseminated histoplasmosis with fever, hepatomegaly; Histoplasma identified from bone marrow biopsy	Recurrent otitis media, streptococcal pharyngitis	Amphotericin B, itraconazole; recurrence 2 years later with abdominal histoplasmosis
	c.289-15T > A	*Histoplasma* spp.	M/4 years (patient 3)	Lymphadenitis	Recurrent otitis media, streptococcal pharyngitis, bronchitis	Amphotericin B, itraconazole
Pedroza et al. (115)	c.233_234 delinsAA, p.S78*	*H. capsulatum*	M/2.5 years, Ecuador	Cutaneous histoplasmosis	*Cryptosporidium parvum* enteritis, oral candidiasis, pneumonia caused by *Pseudomonas aeruginosa* and *Candida albicans*	Amphotericin B for 4 weeks followed by itraconazole prophylaxis
Cabral-Marques et al. (116)	c.345_402del	*Paracoccidioides brasiliensis*	M/11 years, Sao Paulo, Brazil	Prolonged fever and cough, mediastinal lymphadenopathy, bone marrow hypoplasia and tuberculoid granuloma	PCP, recurrent otitis media, and sinopulmonary infections	Treated with 8 months of itraconazole and recovered
Kamchaisatian et al. (118)	Complex mutation in exon 5	*Talaromyces marneffei*	M/14 months, Northeastern Thailand	Prolonged fever, cough, neck pain and bloody sputum; neck imaging showed prevertebral soft tissue swelling. Throat swab, sputum, blood and bone marrow cultures yielded *T. marneffei*	Recurrent pneumonia, oral ulcers, cyclical neutropenia	Treated with amphotericin B for 21 days, followed by itraconazole for 10–12 weeks
	Not stated	*T. marneffei*	M/1 year, Northern Thailand	Fever, cough, dyspnea, lymphadenopathy and pleural effusion; lymph node biposy yielded *T. marneffei*	PCP	Treated with amphotericin B for 21 days, followed by itraconazole for 10–12 weeks
Sripa et al. (119)	Not stated	*T. marneffei*	M/3 years, Thailand	Pneumonia, positive *T. marneffei* culture from tracheal aspirate	PCP, cyclical neutropenia	Treated with itraconazole with good response
Liu et al. (120)	g.IVS1-3T > G	*T. marneffei*	M/2 years, China	Disseminated *T. marneffei* infection with airway granuloma, hepatosplenomegaly and fungemia	BCG-itis, pneumonia	Died of multi-organ failure

*Location of residence is indicated wherever information is available*.

*PCP, Pneumocystis jiroveci pneumonia*.

One patient with NEMO deficiency was reported to have persistent nodal histoplamosis at the age of 52 years. Symptoms including worsening dyspnea and intermittent night sweats that lasted for 1 year, and imaging studies revealed perihilar mass and mediastinal lymphadenopathy. Positive culture of *H. capsulatum* was obtained from paratracheal lymph node biopsy. He responded well to posaconazole (86). A patient with DOCK8 deficiency had miliary pneumonia caused by *H. capsulatum*. She also had numerous infectious caused by viruses (molluscum contagiosum, recurrent herpes zoster, cutaneous human papilloma virus infection), and dermatitis with *Staphylococcus aureus* superinfection (86, 121).

### GATA2 Deficiency

A syndrome of monocytopenia with susceptibility to non-tuberculous mycobacterial (NTM) infections, often termed “MonoMAC,” is caused by haploinsufficiency of the hematopoietic transcription factor GATA2 (122). Majority of the patients have monocytopenia, natural killer (NK), and B lymphocytopenia, while CD4 lymphocytopenia and neutropenia are also common but less marked. Affected individuals are susceptible to a broad range of viral (human herpes virus and human papillomavirus), disseminated NTM, bacterial, and fungal infections (123, 124). In a cohort of 57 patients with *GATA2* mutations evaluated at the National Institutes of Health in the US (124), severe fungal infections were observed in 16%, including invasive aspergillosis (9%), disseminated histoplasmosis (5%), and recalcitrant mucosal candidiasis (5%). Another patient with GATA2 deficiency and disseminated histoplasmosis was reported by Lovell et al. (86). All the patients diagnosed to have GATA deficiency with disseminated histoplamosis were adults (86, 124). Apart from infections, other clinical features of GATA2 deficiency include congenital lymphedema, pulmonary alveolar proteinosis, and predisposition to myelodysplastic syndrome or acute myeloid leukemia, but considerable clinical heterogeneity exists.

### Inborn Errors of IFN-γ-Dependent Immunity

Host defense against intracellular bacterial and fungal pathogens depends on effective cell-mediated immunity, which is coordinated by APC and T-lymphocytes (89, 90, 125). Following phagocytosis, macrophages, monocytes, and DCs secrete IL-12p70, a heterodimer of IL-12p40 (IL12B) and IL-12p35 (IL-12A) that stimulates T and NK cells through its receptor IL-12R, a heterodimer of IL-12Rβ1 and IL-12Rβ2. IL-12Rβ1 is bound to tyrosine kinase 2 (TYK2), and IL-12Rβ2 is bound to Janus kinase-2 (JAK2). IL-12 receptor signaling induces phosphorylation, dimerization, and nuclear translocation of signal transducer and activator of transcription-4 (STAT4) to induce IFN-γ production in T-cells and NK cells, and drives Th1 polarization of CD4+ T-cells. Binding of IFN-γ to its heterodimeric receptor consisting of IFN-γR1 and IFN-γR2 leads to signal transducer and activator of transcription 1 (STAT1) phosphorylation by Janus kinase 1 (JAK1) and JAK2. The phospho-STAT1 (p-STAT1) homodimer translocates to the nucleus and modifies gene expression regulated by the γ-regulated sequencing, resulting in phagocyte activation including production of bactericidal ROS by NADPH oxidase, further IL-12 production, and killing of intracellular pathogens (126, 127). IL-12 production is augmented by a T-cell dependent pathway through interaction of CD40 on the surface of APC with CD40L expressed on activated T-cells (128). The signaling pathway is shown in Figure 2B.

IL-23 shares the p40 component with IL-12p70, and IL-12Rβ1 combines with IL-23 receptor (IL-23R) to form the IL-23R complex (129). IL-23R signaling leads to STAT3/STAT4 heterodimer phosphorylation by TYK2 and JAK2, supporting the proliferation of Th17 cells, which are critical mediators of immunity at the mucosal surface (130). Activated Th17 cells produce IL-17 and IL-22, which induce antimicrobial peptide production in epithelial cells, and the recruitment and activation of inflammatory cells, especially neutrophils (131). These effector functions are critical in the control of mycobacteria, fungi, and bacterial pathogens such as *salmonella* (131–133).

Genetic defects of the IFN-γ-dependent immunity are collectively known as the Mendelian susceptibility to mycobacterial disease (MSMD) (126, 127). These disorders encompass defects of IFN-γ production or response to IFN-γ, caused by mutations in *IL12B, IL12RB1, ISG15, NEMO, IFNGR1, IFNGR2, STAT1, NEMO, IRF8*, and *CYBB*. Altogether, they constitute 18 genetic etiologies of MSMD based on the mode of inheritance, complete or partial defect, expression of the mutant allele, and the functional aberrations. Mycobacterial infection is the sole infectious phenotype in some of these disorders (AD IRF8 deficiency, AR ISG15 deficiency), while others have increased susceptibility to a broader range of pathogens (126, 127). Endemic fungal infections have been reported in AR IL12Rβ1, AR IFN-γR1, and AD GOF STAT1 defects.

#### AR IL12Rβ1 Deficiency

Autosomal recessive IL12Rβ1 deficiency is the most common form of MSMD, accounting for approximately half of the cases in which a genetic cause has been identified (126, 127, 134). Patients with IL12Rβ1 deficiency are recognized by their susceptibility to mycobacterial infections (*M. bovis BCG*, NTM, and *M. tuberculosis*) and non-typhoidal salmonellosis of unusual severity or frequency, but some patients are also susceptible to *Candida, Klebsiella, Nocardia, Leishmania, Histoplasma, Coccicioides, and Paracoccidioides* (134). Peripheral blood mononuclear cells of these patients do not respond to IL-12 and IL-23, resulting in impaired IFN-γ production by T and NK cells. The development of IL-17-producing T-cells is also impaired, due to defect of the IL-23R complex, which is composed of IL-12Rβ1 (135–137). This accounts for the susceptibility to develop CMC observed in 23% of patients (134–137).

The seven reported cases of coccidioidomycosis, PCM, and histoplasmosis in patients with IL12Rβ1 deficiency are summarized in Table 5 (134, 138–141). The age at which disseminated mycoses developed varied from childhood to adulthood, some with past history of mycobacterial infection and salmonellosis. Two cases had recurrence disease, but could be controlled by antifungal treatment.

**Table 5:** Endemic mycoses in IL12RB1 deficiency and INFGR1 deficiency.

	Genetic defect	Endemic fungal pathogen	Gender/age, residence	Clinical manifestations	Other infections and comorbidities	Treatment and outcome
**IL12RB1 deficiency**						
Moraes-Vasconcelos et al. (138)	Homozygous p.L77F	*Paracoccidioides brasiliensis*	M/24 years, Brazil	20 years: fever, hepatosplenomegaly, generalized lymphadenpathy	BCG cervical adenopathy at 7 m, relapse at 2 years 6 years; disseminated non-typhoidal salmonellosis which lasted for 7 years	Treated with trimethoprim-sulfamethoxazole for 5 years with clinical resolution
de Beaucoudrey et al. (137)	Homozygous p.R521X	*Histoplasma* spp.	F/5 years	Disseminated histoplasmosis	Tuberculosis	Not mentioned
Vinh et al. (139)	Homozygous p.C186Y	*Coccidioides* spp.	Patient 1: F/22 years, US	Diffuse lymphadenopathy (cervical, supraclavicular, hilar, mediastinal, retroperitoneal)	Non-typhoidal salmonellosis (bacteremia and lymphadenopathy)	Fluconazole for 1.5 years without recurrence
			Patient 2 (brother of Patient 1): M/6 years, Arizona, US	6 years: coccidioidal pneumonia 14 years: right supraclavicular lymphadenopathy and a nasal lesion 16 years: osteomyelitis of the right proximal tibia	Nil	Received fluconazole for 2 years, developed osteomyelitis 2 months after stopping fluconazole, treated with itraconazole with improvement
Hwangpo et al. (140)	IL12-receptor defect (by functional studies)	*Histoplasma* spp.	M/8 years	Disseminated histoplasmosis with miliary infiltration of the lungs, mediastinal lymphadenopathy, splenomegaly	Not mentioned	Itraconazole
Falcão et al. (141)	Homozygous p.R283X	*Histoplasma capsulatum*	M/4 years, Brazil	4 years: fever, hepatosplenomegaly, generalized lymphadenopathy, bone marrow involvement 6 years: CNS histoplasmosis complicated by hydrocephalus	Tuberculous adenitis	Antifungal treatment and itraconazole prophylaxis
			Brother of the proband	Disseminated histoplasmosis	Tuberculous adenitis, disseminated salmonellosis	Not mentioned
**IFNGR1 defect**						
Zerbe and Holland (146)	Heterozygous c.818del4	*H. capsulatum*	M/3 years, Tennessee, US	3 years: fever, pneumonia, hepatosplenomegaly, cervical and paratracheal lymphadenopathy 4.5 years: fever, pneumonia, generalized lymphadenopathy, sinusitis; lymph node biopsy yielded *H. capsulaum* 4.8 years: right paranasal mass and osteomyelitis of the facial bones requiring debridement	Coexisting MAC infection: 3 years: MAC found in gastric aspirate 7 years: cervical lymphadenopathy and osteomyelitis of rib, biopsy yielded MAC	Repeated courses of intensive antifungal and antimycobacterial therapy, subcutaneous IFN-γ injection led to clearing of all bone lesions. Remained well on prophylactic itraconazole, azithromycin, and IFN-γ
Vinh et al. (147)	Heterozygous c.818del4	*Coccidioides* spp.	M/11 years, Arizona, US	Lobar pneumonia, mediastinal and hilar lymphadenopathy; later developed osteomyelitis involving the vertebral spine and pelvic bone	*Mycobacterium chelonae* pulmonary infection at 11 months; *M. kansasii* abscess involving the cervical spine and retropharyngeal space	Refractory coccidioidomycosis with progressive skeletal lesions despite prolonged use of antifungal therapy (amphotericin B and azoles). Surgical debridement with implantation of amphotericin B-impregnated beads. Adjunctive IFN-γ injection for *M. kansasii* infection with good response
Lee and Lau (manuscript in preparation)	Homozygous c.182dupT, p.V61fs	*Talaromyces marneffei*	F/5 months, Chiang Mai, Thailand	Generalized papular skin lesions, hepatosplenomegaly, osteolytic lesions in the skull, fungemia	11 months: fulminant salmonella septicemia	*T. marneffei* infection resolved with amphotericin B followed by oral itraconazole; died of salmonellosis and massive lower gastrointestinal bleeding

*Location of residence is indicated wherever information is available*.

*IFN, interferon; MAC, Mycobacterium avium complex*.

#### AR IFNγ Receptor Deficiency

IFN-γR1 and IFN-γ2 are the ligand-binding and transducing receptor chains of the INF-γ receptor, respectively. Biallelic null mutations in the *IFNGR1* gene result in AR complete IFNγR1 deficiency, which is characterized by high plasma concentration of IFN-γ and a lack of response to IFNγ *in vitro* (142, 143). These patients have early onset, life-threatening disseminated mycobacterial infections and the overall prognosis is poor with high rate of fatality. Other infections caused by viruses (cytomegalovirus, respiratory syncytial virus, varicellar zoster virus, human herpes virus 8) and bacteria (*Listeria monocytogenes*) have been described. In AR partial IFN-γR1 deficiency, the clinical phenotype is less severe (144). Apart from mycobacterial infections, bacterial, viral, and parasitic organisms have been reported (126, 127, 142, 144).

Autosomal dominant partial IFN-γR1 deficiency is caused by mono-allelic mutations affecting exon 6 and exon 7. A hotspot mutation, 818del4, accounts for over 80% of patients with AD IFN-γR1 deficiency (126, 127). In contrast with AR IFN-γR1 deficiency, these is an increase in IFN-γR1 protein expression on the cell surface, due to the accumulation of truncated IFN-γR1 receptors lacking the recycling domain. Despite the presence of receptors encoded by the wild-type *IFNGR1* allele, the non-functioning IFN-γR1 protein impedes the normal function of IFN-γR1 dimers by negative dominance and impairs the response to IFN-γ *in vitro* (145). The clinical features are less severe than those seen in patients in AR complete IFN-γR1 deficiency. Most patients have BCG or NTM infections, and salmonella infection was reported in only 5% of cases (126, 127, 142). Disseminated histoplasmosis (146) and coccidioidomycosis (147) were reported in two patients with IFN-γR1 deficiency and both had a refractory or relapsing course (Table 5). Our group diagnosed AR IFN-γ receptor 1 deficiency in a Burmese infant suffering from disseminated *T. marneffei* infection, and she eventually died of salmonellosis (manuscript in preparation).

IFN-γR2 deficiency is less common than IFN-γR1 deficiency. Similarly, AR complete or partial IFN-γR2 deficiency cause increased susceptibility to mycobacterial infections (148–150); other infections are rare and include salmonellosis in one patient and cytomegalovirus disease in three patients (126), but mycosis has not been reported. AD form of partial IFN-γR2 deficiency was diagnosed in a patient with mild BCG disease, and clinical penetrance is very low (151).

#### AD STAT1 Defect

Signal transducer and activator of transcription 1 (STAT1) is a transcription factor involved in cellular responses mediated by type I (IFNα/β), type II (IFN-γ), and type III (IFN-λ) IFNs (152). AR complete STAT1 deficiency is characterized by the absence of STAT1 expression and abolished cellular response to IFN-γ as well as IFN-α/β and IFN-λ, resulting in severely impaired antimycobacterial and antiviral immunity (153, 154). Patients with complete STAT1 deficiency caused by null mutations have increased susceptibility to mycobacterial, viral, and bacterial infections, whereas biallelic hypomorphic mutations in AR partial STAT1 deficiency are associated with milder clinical severity (153–155). AD partial STAT1 deficiency with mono-allelic loss-of-function (LOF) *STAT1* mutation predispose to mycobacterial infection (156); in contrast, AD GOF *STAT1* mutation is recognized as the most common cause of CMC disease, accounting for half of the cases (157–159). Majority of the mutations affect the coiled-coil domain or DNA-binding domain of STAT1 (159). They increase STAT1 phosphorylation by impairing nuclear dephosphorylation. They are GOF for the STAT1-dependent cytokines including IFN-α, IFN-β, IFN-γ, and IL-27, which repress Th17 development, accounting for the low numbers of IL-17-producing T-cells in these patients (160).

In addition to CMC, invasive mycoses caused by a variety of yeasts (e.g., *Cryptococcus*), molds (*Aspergillus, Fusarium*), and endemic fungi (*Histoplasma, Coccidioides, T. marneffei*) have been reported in patients with GOF *STAT1* mutations, as summarized in Table 6 (161, 162). The two patients who developed disseminated coccidioidomycosis during childhood had progressive disease, which persisted into teenage despite intensive treatment; one had spinal cord compression and one died of overwhelming coccidioidomycosis at 17 years. Of note, the latter patient did not have CMC or other unusual infections, implying that coccidioidomycosis could be the sole infection in patients with GOF STAT1 defect. Three patients had disseminated histoplasmosis and two of them had recurrent disease. All of them responded well to antifungal treatment (161).

**Table 6:** Endemic mycoses in AD signal transducer and activator of transcription 1 defect.

	Genetic defect	Endemic fungal pathogen	Gender/age, residence	Clinical manifestations	Other infections and comorbidities	Treatment and outcome
Sampaio et al. (161)	Heterozygous p.E353K	*Coccidioides* spp.	Patient 1: F/17 years, AZ, USA	Coccidioidal pneumonia, mediastinal lymphadenopathy, and sternocleidomastoid abscess; progressive disease with osteomyelitis of the vertebral spine and lesions in the skin, liver, and spleen at 20 years	Extensive persistent tinea capitis and kerion caused by *T. tonsurans*	Progressive disease despite prolonged antifungal therapy including amphotericin B, azoles and caspofungin. Developed spinal cord compression at 20 years due to intramedullary lesion
	Heterozygous p.A267V	*Coccidioides immitis*	Patient 2: F/9 years, AZ, USA	Coccidioidal pneumonia and intrathoracic lymphadenopathy, osteomyelitis of the vertebral spine; progressive disease with CNS involvement, lymphadenopathy, retinal mass, and multifocal osteomyelitis	Nil	Progressive disease despite prolonged antifungal therapy including amphotericin B, azoles, and caspofungin, suboptimal response to adjunctive IFN-γ therapy. Died of overwhelming coccidioidomycosis at 17 years
	Heterozygous p.T385M	*Histoplasma capsulatum*	Patient 3: M/21 years	Disseminated histoplasmosis at 12 years	CMC, *M. fortuitum* cervical lymphadenopathy, recurrent pneumonia and herpes zoster, bronchiecatasis Recurrent fractures, progressive bilateral upper limb muscle atrophy	Histoplasmosis treated with itraconazole with good response
	Heterozygous p.R274G	*H. capsulatum*	Patient 4: M/31 years	17 years: disseminated histoplasmosis presenting with fever, weight loss, lymphadenopathy with liver, and bone marrow involvement 30 years: CNS histoplasmosis	CMC, warts, recurrent *Salmonella* septicemia Type 1 DM at 24 years 31 years: PML caused by JC virus	Histoplasmosis treated with amphotericin B for 6 months followed by fluconazole with multiple relapses that responded to intensified treatment
	Heterozygous p.F172L	*H. capsulatum*	Patient 5: F/25 years	7 years: disseminated histoplasmosis presenting with fever, hepatosplenomegaly, lymphadenopathy, and dyspnea; recurrence at 8 years	CMC Subclinical hypothyroidism at 14 years, ovarian failure at 24 years	Histoplasmosis treated with itraconazole with good response
Lee et al. (162)	Heterozygous p.A267V	*Talaromyces marneffei*	Patient 1: M/14 years, Hong Kong	Disseminated *T. marneffei* infection at 15 years with generalized lymphadenopathy, positive culture of *T. marneffei* from lymph node biopsy	CMC	Amphotericin B, itraconazole prophylaxis
	Heterozygous p.L358F	*T. marneffei*	Patient 2: F/8 years, Hong Kong	Cavitating pneumonia with cystic cavities; mediastinal and hilar lymphadenopathy, positive culture of *T. marneffei* from BAL CMC	Recurrent sinopulmonary infections Influenza A (H1N1)	Liposomal amphotericin, itraconazole prophylaxis
	Heterozygous p.T288I	*T. marneffei*	Patient 3: F/16 years, Hong Kong	Cervical lymphadenopathy, positive culture of *T. marneffei* and *M. tuberculosis* from lymph node biopsy; concomitant axillary, mesenteric, and retroperitoneal lymphadenopathy	CMC Recurrent sinopulmonary infections and herpes zoster EBV-associated HLH Disseminated aspergillosis	*T. marneffei* infection responded well to itraconazole Disseminated aspergillosis and EBV-associated HLH at 16 years, died of massive gastrointestinal hemorrhage
Lee and Lau, unpublished	Heterozygous p.M390I	*T. marneffei*	M/40 years, Hong Kong	10 years: cervical lymphadenopathy complicated by ulcerations; perforation of the hard palate, mediastinal lymphadenopathy causing SVC obstruction and sternal erosion, tissue culture yielded *T. marneffei* 17 years: osteomyelitis of the thumb, forearm, and tibia	CMC	Relapsing and remitting disease course on prolonged treatment of amphotericin B and fluconazole till 20 years; infection cleared with residual scarring of the skin and dilated veins on the chest

*Location of residence is indicated wherever information is available*.

*BAL, bronchoalveolar lavage; CMC, chronic mucocutaneous candidiasis; CNS, central nervous system; DM, diabetes mellitus; EBV, Epstein–Barr virus; IFN, interferon; PML, progressive multifocal leukoencephalopathy; SVC, superior vena cava*.

In Hong Kong, five pediatric patients were diagnosed to have *T. marneffei* infection from 1983 to 2009 in a single center (74, 85, 162, 163). One patient was lost to follow-up after complete recovery from *T. marneffei* infection (163), while the remaining four patients underwent thorough immunological investigations and genetic studies, and all were found to have GOF STAT1 defect (162). They all had CMC, and two had recurrent sinopulmonary infections, herpes virus infections (cytomegalovirus, Epstein–Barr virus and varicella zoster), TB, and disseminated aspergillosis (85, 162). The patient with chronic relapsing *T. marneffei* infection first reported by Yuen et al. in 1986 was subsequently investigated when his son was referred for CMC, and both were confirmed to have AD GOF STAT1 defect in 2015.

An increased incidence of herpes virus infections as well as TB and NTM infections (e.g., *M. bovis BCG*) has been observed in patients with GOF STAT1 defect, and it was thought that the enhancement of signaling downstream to IFN-α/IFN-β and IFN-γ caused by GOF *STAT1* mutations could lead to exhaustion of virus-specific T-cells and refractory response to IFN-γ. Other clinical manifestations of GOF *STAT1* mutations include autoimmunity (e.g., type 1 diabetes mellitus, thyroid disease, autoimmune cytopenia, and hepatitis), vascular aneurysms, and malignancies, particularly squamous cell carcinoma of the esophagus (159, 160, 164).

### Inborn Errors of Immunity Associated with Impaired Th-17-Mediated Immunity

Autosomal-dominant hyper-IgE syndrome caused by LOF *STAT3* mutation, also known as Job’s syndrome, is characterized by recurrent staphylococcal cold abscesses, pneumonia, and eczema. In addition, patients often display joint hyperextensibility, skeletal abnormalities and pathological fractures, delayed dental deciduation, coronary artery aneurysms, brain lesions, and Chiari’s malformation (165, 166). Pneumonia is typically caused by *S. aureus, Haemophilus influenzae*, or *Streptococcus pneumoniae*, and is often complicated by pneumoatocele formation. Approximately 20% of patients with AD hyper-IgE syndrome develop invasive infection caused by Aspergillus, which has angioinvasive properties with tendency to cause hematogenous dissemination (167–170). STAT3 promotes the expression of the gene encoding the retinoic acid receptor-related orphan receptor-γt (RORγt), and important transcription factor that drives differentiation of naïve CD4+ T-cells to Th17 cells. Dominant negative *STAT3* mutation leads to impaired RORγt induction in response to IL-1β, IL-6, and IL-23, causing severe deficiency of IL-17-producing effector cells (171, 172).

Three cases of coccidioidomycosis, 10 cases of histoplasmosis and two cases of *T. marneffei* infection were reported in patients with hyper-IgE syndrome, as summarized in Table 7 (85, 87, 173–183). The three cases of hyper-IgE syndrome with coccidioidomycosis all had CNS involvement (87, 173, 174), while those with IL-12Rβ1 deficiency (139), IFN-γR1 deficiency (147), and GOF STAT1 defect (161) mainly had lymphadenopathy and osteomyelitis, so it appears that there is a predilection for *Coccidioides* to disseminate to the CNS in hyper-IgE syndrome. In contrast, disseminated histoplasmosis occurred in IL-12Rβ1 deficiency (141), IFN-γR1 deficiency (146), and GOF STAT1 defect (161), but 7 out of 10 cases of histoplasmosis in hyper-IgE syndrome involved the aerodigestive tract only (175–182). Two cases of *T. marneffei* infection were reported in hyper-IgE syndrome (85, 183).

**Table 7:** Endemic mycoses in AD hyper-IgE syndrome (Job syndrome).

	Genetic defect	Endemic fungal pathogen	Gender/age, residence	Clinical manifestations	Other infections and comorbidities	Treatment and outcome
Stanga and Dajud (173)	Not stated	*Coccidioides immitis*	F/4 years	Coccidioidal meningitis with cerebral infarct at multiple sites, gross left hemiplegia	Recurrent sinus and skin infections, eczema	Treated with amphotericin B and fluconazole, minimal residual deficits
Powers et al. (174)	Heterozygous p.T412S	*C. immitis*	F/17 years, UT, USA	Coccidioidal meningitis and cerebral abscess, altered mental status requiring temporary intubation	*Staphylococcus aureus* skin and soft tissue infections, recurrent sinopulmonary infections	Improved with liposomal amphotericin, followed by fluconazole prophylaxis
Odio et al. (87)	Heterozygous p.V713M	*C. immitis*	F/4 years, AZ, USA	Coccidioidal meningitis and pulmonary infection presenting with fever, headache, and seizure	Recurrent pneumonia and otitis, skin infections, eczema, thrush	Complicated by cerebral vascular accident and hydrocephalus, treated with liposomal amphotericin and fluconazole Residual left hemiparesis
Alberti-Flor et al. (175)	Not stated	*Histoplasma capsulatum*	M/16 years	Histoplasmosis with ileocecal involvement	Not stated	Resection of terminal ileum and right colon, treated with ketoconazole with good response
Cappell et al. (176)	Not stated	*H. capsulatum*	F/27 years	Disseminated histoplasmosis with cecum, colon, and bone marrow involvement	Not stated	Treated with amphotericin B and ketoconazole
Desai et al. (177)	Not stated	*H. capsulatum*	M/33 years	Histoplasmosis with pulmonary and tongue involvement	Eczema, recurrent sinopulmonary infections, thrush and onychomycosis, *Staphylococcal* septic arthritis, *Cryptococcus* meningitis at 37 years	Histoplasmosis treated with amphotericin B and ketoconazole; lobectomy for bronchiectasis
Steiner et al. (178)	Not stated	*H. capsulatum*	F/14 years	Histoplasmosis with ileocecal involvement	Staphylococcal pneumonitis complicated by cystic changes and bronchopleural fistula	Treated with 12 months of itraconzole with good response
Robinson et al. (179)	Heterozygous p.K591M	*H. capsulatum*	M/33 months	Disseminated histoplasmosis with pneumonia and hepatosplenomegaly	Pneumonia, otitis, thrush, eczema, folliculitis, gastroenteritis, multiple fractures, pneumatocele, multiple allergy, developmental delay	Not stated
Rana et al. (180)	Not stated	*H. capsulatum*	F/4 years, India	Histoplasmosis with rectal involvement	Recurrent subcutaneous abscess, giardiasis, *Entamoeba* infection, molluscum contagiosum, milk allergy	Good response to treatment
Jiao et al. (181)	Not stated	*H. capsulatum*	M/21 years	Terminal ileal perforation, histopathology showed *H. capsulatum* within histiocytes	Not stated	Partial small bowel resection Liposomal amphotericin, itraconazole
Odio et al. (182)	Heterozygous p.V432M	*H. capsulatum*	M/10 years, US	Disseminated histoplasmosis with pulmonary, liver, and spleen involvement	Not stated	Treated with liposomal amphotericin, itraconzaole, and posaconazole
	Heterozygous p.F621V	*H. capsulatum*	F/15 years, US	Histoplasmosis with gastrointestinal involvement, complicated by duodenal stricture	Not stated	Treated with liposomal amphotericin and itraconazole
	Heterozygous p.W479C	*H. capsulatum*	F/22 years, US	Histoplasmosis with laryngeal involvement requiring reconstructive largyngoplasty	Not stated	Treated with ketoconazole
Ma et al. (183)	Not stated	*Talaromyces marneffei*	M/30 years, Hong Kong	Lung abscess, massive hemoptysis	*Stenotrophomonas maltophilia* lung abscess, recurrent pneumonia, skin infections	Treated with amphotericin B, died of respiratory failure due to rapid disease progression
Lee et al. (85)	Heterozygous p.D374G	*T. marneffei*	F/12 months, Guangzhou, China	Disseminated *T. marneffei* infection with pancytopenia and hepatosplenomegaly, positive culture of *T. marneffei f*rom bone marrow	Pulmonary aspergillosis, *Staphylococcus* septicemia, pneumatocele, and pneumothorax	*T. marneffei* infection treated with itraconazole with good response

*Location of residence and mutation of STAT3 gene are provided wherever information is available*.

To summarize, the susceptibility to endemic mycoses in CD40L deficiency, IL12Rβ1 deficiency, and IFN-γR1 deficiency highlights the critical role of the IL-12/IFN-γ crosstalk in macrophage activation and killing of these endemic fungi, while the deficiency of Th17 cells in patients with GOF STAT1 defect and AD hyper-IgE syndrome puts them at risk for both CMC and IFIs, and they frequently have CMC due to impaired mucosal immunity against *C. albicans*. Defective oxidative burst alone, as in CGD, is not sufficient to cause increased risk to endemic mycoses, suggestive that other mechanisms of phagosomal killing may compensate for the lack of NADPH oxidase activity to control these endemic fungi, distinguishing them from many other invasive fungi to which CGD patients are susceptible.

## Protective Immunity Against Endemic Mycoses Conferred by Cytokines: Insights from Biologics and Anti-IFN-γ Autoantibodies

The development of biologic response modifiers (BRMs), such as monoclonal antibodies and receptor antagonists that target pro-inflammatory cytokines and their receptors has led to major advances in the treatment of autoimmune and malignant disorders. However, they have the potential to suppress host immune response and increase the risk of infections. The use of biologics is associated with a small but important risk of IFI. Histoplasmosis is the most common IFI associated with TNF-α inhibitors (10, 59, 184, 185). In a survey of infectious disease specialists, histoplasmosis was second only to *S. aureus* as the cause of serious infection complicating anti-TNF and other BRM (184). In most cases, patients reside in areas where the fungus is endemic and have received other immunosuppressants concurrently. Up to 2% of patients receiving BRMs will develop coccidioidomycosis if they reside in an endemic region (186). The American Academy of Pediatrics (AAP) Committee on Infectious Diseases recommends that patients on BRM should be enquired about epidemiologic risk factors and possible exposures to histoplasmosis and coccidioidomycosis, which have symptoms and signs that significantly overlap with TB. If there is suspicion of signs or symptoms compatible with acute histoplasmosis or coccidioidomycosis, BRM should be discontinued immediately and patients will require evaluation with a combination of chest radiography and serologic, antigen detection, and culture tests, which are best conducted in consultation with an infectious diseases expert (186, 187).

Other targeted therapies have also been implicated as risk factors for endemic mycoses. In Hong Kong, four cases of disseminated *T. marneffei* infection were diagnosed in adult hematology patients receiving anti-CD20 monoclonal antibodies (rituximab and obinutuzumab) and kinase inhibitors (84, 188, 189). The observation is revealing, as the importance of B-lymphocytes and humoral immune response against fungus is not well defined, and *T. marneffei* infection has so far not been reported in patients with congenital agammaglobulinemia. Depletion of B-lymphocytes may lead to profound deficiency in the production of neutralizing antibodies against key virulence factors of *T. marneffei*. Kinase inhibitors such as ruxolitinib and sorafenib are increasingly used in treating hematological malignancies, solid tumors, psoriasis, and alopecia areata. Ruxolitinib is a selective JAK1 and JAK2 inhibitor that interferes with the IFN-γ and its downstream JAK-STAT signaling. Sorafenib is a multi-kinase inhibitor that exhibits immunomodulatory effect by impairing T-lymphocyte proliferation, production of IFN-γ and other pro-inflammatory cytokines, NK cell, and DC functions. The suppression of IFN-γ signaling pathway poses risk to develop *T. marneffei* infection (84). It would be important for clinicians to have a high index of suspicion on *T. marneffei* infection in patients receiving these targeted therapies to avoid delay in diagnosis and treatment.

Autoantibody against IFN-γ has been reported to be associated with adult-onset immunodeficiency in patients from Asian countries (190–195). Disseminated NTM is the most common clinical presentation. In a cross-sectional, case-control study conducted in Chiang Mai, Thailand showed that patients with opportunistic infections including disseminated NTM, disseminated *T. marneffei* infection, melioidosis and non-typhoidal Salmonellosis had anti-IFN-γ autoantibody level above 99th percentile of cut-off for healthy individuals, and the level of autoantibody in patients who had active opportunistic infection was relatively higher than those without active infection (193). Similar observations were also reported in Hong Kong and Taiwan (190, 192, 194, 195). HLA class II molecules HLA-DRB1*15:02–HLA-DQB1*05:01 and HLA-DRB1*16:02–HLA-DQB1*05:02 are specifically associated with anti-IFN-γ autoantibodies and NTM (196, 197). The high frequency of such alleles in Southeast Asia might account for the relatively high prevalence this condition in the Asian population. The study by Lin et al. showed that anti-IFN-γ autoantibody from patients recognizes an epitope at the C terminus of IFN-γ, and binding of the autoantibody neutralizes IFNγ-induced signaling. This epitope displays a high degree of sequence homology to the Aspergillus Noc2 protein. It was postulated that in the warm and humid environment of Southeast Asia where exposure to Aspergillus species is common in everyday life, some individuals might develop anti-IFN-γ autoantibodies due to molecular mimicry (198). The co-evolution of anti-IFN-γ autoantibody production as the susceptibility trait amongst Southeast Asians, and the high prevalence of *T. marneffei*, NTM, and mellioidosis in this region is a unique combination not observed in the rest of the world, and it is so interesting that exposure to a common environmental fungal agent could indirectly induce susceptibility to other pathogens.

## Future Perspectives

The understanding about inborn errors of immunity predisposing to endemic mycoses is limited. First, exposure to these environmental fungal pathogens is often a chance event that depends on the natural habitat and climate, as well as the circumstances and activities in which the individual is engaged. Thus, the number of cases of endemic mycoses associating with PID is likely to be low. The proportion of patients with PID amongst those with disseminated endemic mycoses is unknown, due to the lack of information about the population incidence (i.e., the “denominator”). Second, some forms of endemic mycoses, particularly *T. marneffei* are prevalent in less resourced countries where well-developed clinical service for PID is lacking, and diagnosis is often delayed or missed. Third, the global health impact of these geographically restricted endemic fungi is probably less than those opportunistic fungal pathogens of worldwide distribution causing high disease burden in immunocompromised patients (e.g., *Candida, Aspergillus, P. jiroveci, Cryptococcus*, and *Rhizopus*), and they probably generate less attractions and interests in the global public and scientific community (7, 199, 200). Regional and global effort in establishing registries on disseminated endemic mycoses is crucial, in order to collect patient demographic data and determine their true population incidence.

While endemic mycoses are geographically restricted to certain regions, clinicians looking after patients with PID or acquired immunodeficiencies should gain knowledge about these rare fungal infections so that appropriate advice can be given to their patients when planning for travels, and to have heightened awareness of such diagnostic possibility when they return from endemic areas. Climate, environment, and exposure (behavior) are the “triad” that determine the risk of endemic fungal infection in susceptible hosts. High-risk activities that increase the chance of exposure to fungal conidia should be avoided, otherwise, precautionary measures should be taken.

The discovery of PID predisposing to endemic mycoses is a fascinating journey, as it illuminates the key molecules and signaling pathways that are crucial in host defense against this group of dimorphic fungi, which are closely related in phylogeny. As disseminated coccidioidomycosis, histoplasmosis, PCM, and *T. marneffei* infection are recognized as AIDS-defining illness, they should also be regarded as indicator diseases for PID in individuals who are HIV-negative, and without known risk factors and secondary immunosuppression, particularly in children. There is a need to design an algorithm to evaluate such patients, with stepwise immunological investigations. A detailed history on previous infections, CMC, autoimmune manifestations, and family history should be taken. We recommend a basic panel of immunological evaluations including Ig pattern (IgG, IgA, IgM, and IgE), lymphocyte subset, and nitroblue tetrazolium, or dihydrorhodamine tests to assess oxidative burst activity. These patients should be assessed by immunologists. Abnormal results obtained during acute illness should be repeated upon full recovery. A systematic approach will facilitate clinicians to identify patients who warrant candidate gene studies or functional delineation of the pathways involved in immune recognition, T-cell activation and differentiation, cytokine signaling, and phagocytic killing. The presence of anti-IFN-γ autoantibody should be excluded. Functional evaluation of the IL-12/IFN-γ axis, STAT1 phosphorylation studies, and Th17 enumeration will be particularly relevant in this context. The utilization of next-generation sequencing techniques may lead to discoveries of novel monogenic disorders causing unique susceptibility to endemic mycoses.

## Author Contributions

PL wrote the article. Y-LL provided the conceptual framework and reviewed the manuscript.

## Conflict of Interest Statement

The authors declare that the research was conducted in the absence of any commercial or financial relationships that could be construed as a potential conflict of interest. The reviewer, PR-J, and handling editor declared their shared affiliation and the handling editor states that the process nevertheless met the standards of a fair and objective review.
